# Associations between parent-adolescent sports participation and depression among Chinese youth: a serial mediation model of the parent-adolescent relationship and academic difficulty

**DOI:** 10.3389/fpsyg.2026.1756418

**Published:** 2026-05-29

**Authors:** Yingchen Bi

**Affiliations:** Department of Physical Education, Beihua University, Jilin City, Jilin Province, China

**Keywords:** academic difficulty, depression, parent-adolescent relationship, parent-adolescent sport participation, serial mediation model

## Abstract

**Background:**

Adolescent depression is a major public health concern, particularly in educational contexts characterized by high academic pressure. This study examined the association between parent–adolescent sports participation (PSP) and adolescent depression, with attention to the serial mediating roles of the parent–adolescent relationship (PR) and academic difficulty (AD).

**Methods:**

Using cross-sectional data from the China Education Panel Survey (CEPS), the analysis included 16,984 early adolescents, including 8,560 males and 8,424 females. PSP, PR, AD, and depression were assessed using self-reported measures. Data were analyzed using Model 6 of the PROCESS Macro in SPSS 26.0.

**Results:**

PSP was negatively associated with depression, *β* = −0.08, *p* < 0.001, 95% CI [−0.10, −0.07]. PR was also negatively associated with depression, *β* = −0.21, *p* < 0.001, whereas AD was positively associated with depression, *β* = 0.18, *p* < 0.001, 95% CI [0.16, 0.19]. The serial indirect association between PSP and depression via PR and AD was statistically significant, indirect effect = −0.01, 95% CI [−0.006, −0.004].

**Conclusion:**

These findings highlight the relevance of family-based sports participation, parent–adolescent relationships, and academic experiences in understanding depression among Chinese adolescents.

## 1 Introduction

Adolescence is a critical developmental stage characterized by significant physical, cognitive, and emotional changes. During this period, adolescents are particularly vulnerable to mental health problems due to social pressures, academic stress, and changes in family dynamics. Among these problems, depression is a major concern because it can negatively affect adolescents’ mental health, social relationships, academic performance, and overall development. Globally, approximately 34% of adolescents experience depressive symptoms (Shorey et al., 2022). In China, a study of 4,153 adolescents found that 27.5% reported depressive moods, indicating that adolescent depression is also a serious public health concern in the Chinese context (Chen et al., 2020). Moreover, adolescent depression is often associated with risky behaviors, such as substance abuse and self-harm, and may co-occur with other mental health disorders, including anxiety, attention deficit hyperactivity disorder (ADHD), and eating disorders (Cybulski et al., 2021; Soleimani et al., 2019; Seligman and Ollendick, 1998; Wang S. et al., 2025; Wang J. et al., 2025). Therefore, identifying factors associated with adolescent depression is important for promoting adolescents’ mental health and development.

Numerous studies have demonstrated that adolescents’ participation in sports activities is associated with lower levels of depression (Oberste et al., 2020; Hong et al., 2009). The relationship between sports activities and depression in adolescents may be influenced by social factors, as sports activities and social support are interdependent. Thus, sports participation may also help foster a positive parent-adolescent relationship (Partridge et al., 2008). For adolescents, participating in sports provides opportunities for social interactions and contributes to their emotional well-being (Kleppang et al., 2018; Vilhjalmsson and Thorlindsson, 1992). Ornelas et al. (2007) reported that parents play a role as a source of social support during adolescence, and that adolescents who participate in sports with their parents display lower depressive symptoms compared to those who do not engage in sports. Consequently, adolescents who participate in sports activities with their parents are likely to experience stronger social bonds with them, which may be associated with lower levels of depressive emotions. However, existing research has predominantly focused on studying the impact of individual physical activity or sports participation on adolescents’ mental health, with limited attention given to parent-adolescent sports participation (PSP) and the mechanisms through which PSP may be linked to adolescent depression (Archer, 2014; Goldfield et al., 2011).

PSP may first be linked to adolescent depression through its association with the parent–child relationship (PR) (Lisinskiene and Lochbaum, 2019). The parent–child relationship refers to the bidirectional connection between parent and child, where the parents’ characteristics, parenting style, educational approach, and attitudes directly influence the child’s mental and physical development (Reitman and Asseff, 2010). Attachment theory provides a useful explanation for why PSP may contribute to a more PR. According to this theory, adolescents develop emotional security through repeated interactions in which parents provide responsiveness, support, and companionship (Fearon and Roisman, 2017). PSP offers such opportunities because it allows parents and adolescents to spend time together, communicate, cooperate, and share common interests in a relatively relaxed setting. Stefansen et al. (2018) argued that parents’ sports participation is an intervention strategy that fosters a positive emotional bond with their children, and engaging in sports together can enhance emotional intimacy through the sharing of common interests. Knoester and Randolph (2019) stated that children who spent time with their fathers every week or more frequently, engaging in sports or other outdoor activities, demonstrated stronger intimacy with their fathers. Therefore, compared with sports participation alone, PSP may have particular relational value, as it may strengthen emotional closeness, trust, and mutual support between parents and adolescents. These relational benefits may be important for alleviating adolescent depression.

Meanwhile, a more positive PR may also play an important role in adolescents’ academic adjustment, particularly when they encounter academic difficulty (AD) (Yang et al., 2024). In general, AD occurs when a student lacks sufficient learning motivation due to family-related, school-related, or social factors, or when they realize their inability to achieve learning goals owing to the use of ineffective learning methods, resulting in poor academic performance (Liu, 2019). Family systems theory offers a theoretical basis for understanding this association. From this perspective, AD adjustment is embedded within the broader family system, and the quality of parent–child interaction may influence how adolescents respond to academic demands (Jeanfreau et al., 2020). A supportive PR can provide emotional security, encouragement, guidance, and problem-solving resources when adolescents encounter academic challenges. By contrast, a distant, conflictual, or unsupportive PR may weaken these resources and make academic setbacks more difficult to manage (Shao and Kang, 2022). Parents’ active interest in their children and a favorable family environment can protect children from experiencing academic and social challenges in school caused by the social maladjustment syndrome (Zimmer-Gembeck et al., 2023). The social support provided by parents, such as encouragement, compliments, comfort, and understanding, is critical for adolescents’ successful academic achievements. Wang et al. (2021) reported that PR is a predictor of academic achievement, where better PR is associated with higher academic performance. Conversely, students who experience dismissal or lack of support from their parents demonstrate poorer academic performance (Jeynes, 2005).

Academic difficulty may further have important implications for adolescents’ emotional adjustment, particularly in educational contexts characterized by intense academic pressure (Kristensen et al., 2023). Chen et al. (1995) stated that the higher level of depression among Chinese youth could be attributed to the stressful academic environment in Chinese schools. Adolescents in East Asian countries are presumed to encounter more academic difficulties compared to their Western counterparts, given the strong emphasis on college entrance in these countries (Lee et al., 2013). When students fail to achieve desired grades or academic goals, they may view AD as a reflection of personal inadequacy and become trapped in self-doubt and negative self-attribution, which can lead to depression (Fröjd et al., 2008). Consistently, Wang S. et al. (2025) and Wang J. et al. (2025) reported a significant positive correlation between AD and depression among Chinese students. From the pressure-buffering perspective, supportive resources may help adolescents cope with the psychological burden imposed by academic difficulty (Cohen and Wills, 1985). In this regard, PSP may be particularly relevant because it may not only provide opportunities for physical activity but also foster supportive parent–child interactions that help adolescents manage academic stress. Taken together, these arguments suggest that PSP may foster a more positive PR, which may help adolescents cope with AD and, in turn, be associated with lower levels of depressive emotions.

**Figure 1:**
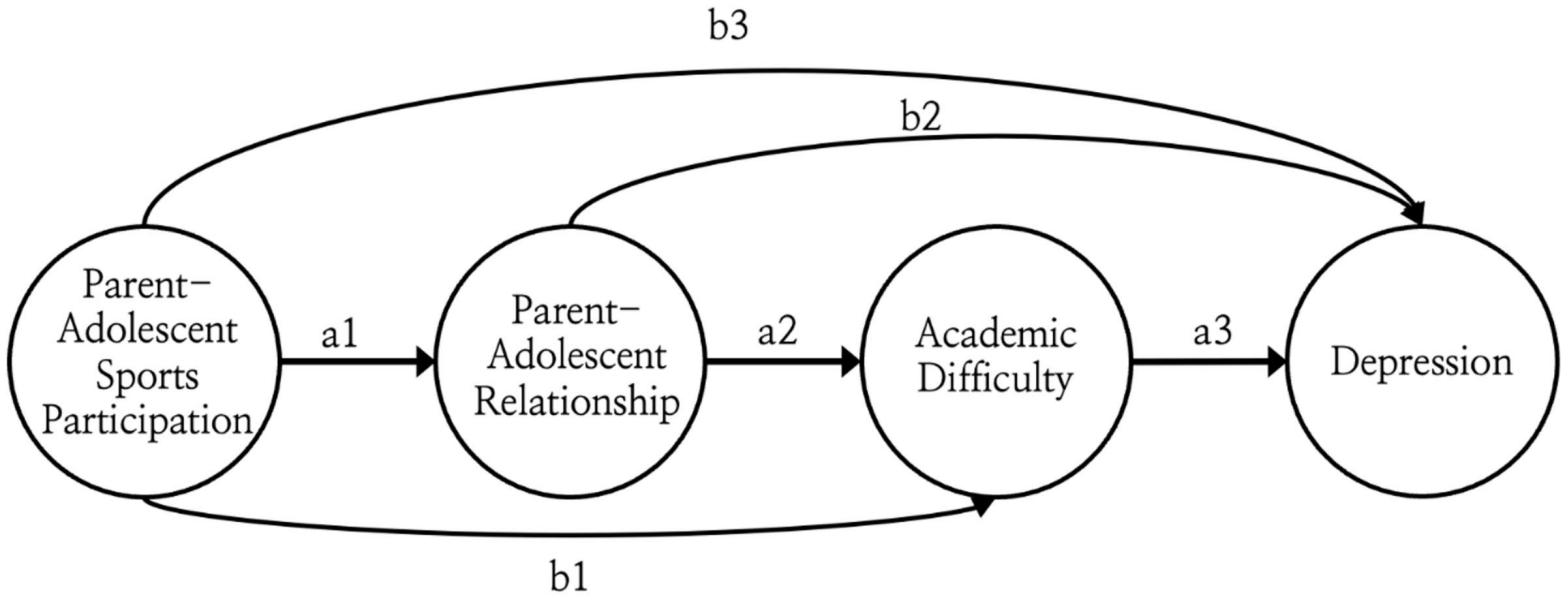
Serial multiple mediation model.

To comprehend the symptoms of depression among adolescents in East Asian culture, which differ from those in Western culture, it is important to consider academic factors. Such an approach can provide insights into the relationship between adolescent depression and personal development within a specific cultural context. Additionally, while prior research has explored the associations among PSP, PR, AD, and depression, there has been relatively little research interest in integrating these factors into a serial mediation model. This study extends previous work by examining how PSP relates to adolescent depression through the sequential processes of PR and AD. In this context, the present study aims to test the serial multiple mediation model, proposing that PSP is associated with adolescent depression through a serial mediation process involving PR and AD in Chinese youth. Figure 1 illustrates the study model.

## 2 Materials and methods

### 2.1 Study data

The data used in this study were obtained from the China Education Panel Survey (CEPS). Launched by the National Survey Research Center in July 2013, CEPS represents the first national-level survey conducted in China for middle school and beyond, employing strict probability sampling. The survey involved the selection of 112 schools and 438 classes through stratified multiple stage sampling and probability-proportional-to-size sampling. For our analysis, we obtained the data of 10,279 seventh graders and 9,207 ninth graders. After excluding missing data, the final dataset comprised 16,984 students (male = 8,560, female = 8,424).

### 2.2 Instruments

#### 2.2.1 Parent- adolescent sports participation (PSP)

PSP was measured using a single question: “How often do you participate in exercise (or sports) with your parents?” The response options were: (1) (never), (2) (less than once a year), (3) (less than once in 6 months), (4) (less than once a month), (5) (less than once a week), and (6) (once or more per week). Higher scores indicated greater PSP frequency.

#### 2.2.2 Parent-adolescent relationship (PR)

PR was evaluated using two questions: (1) “How is your relationship with your father?” and (2) “How is your relationship with your mother?” The responses were assessed separately using a three-point scale (“1 = not close,” “2 = normal,” and “3 = close”). Higher scores indicated better PR. For children with a single parent, only the question pertaining to the existing parent was used, while for children with both parents, the average of the two scores was utilized for the final analysis.

#### 2.2.3 Academic difficulty (AD)

AD was measured using a single question that asked about the survey participants’ perceived difficulty with Chinese, math, and foreign language classes: “How difficult is it for you to learn Chinese, math, and foreign languages?” Participants responded on a four-point scale: “1=Not difficult at all,” “2=Not very difficult,” “3=A bit difficult,” and “4 = Very difficult.” Higher scores indicated greater academic difficulty.

#### 2.2.4 Depression

The survey participants’ depressive mood was assessed by rating the frequency of experiencing five feelings (adjectives) in the past week: frustrated, lonely, unpleasant, the mundanity of life, and sad. Responses were given on a five-point scale: “1 = Never,” “2 = 1–2 days,” “3 = 3–4 days,” “4 = 4–5 days,” “5=Almost every day.” The internal consistency (Cronbach’s *α*) for the five items measuring depressive mood was 0.86.

#### 2.2.5 Control variables

Following the family investment model, we considered family socioeconomic status (SES) and parents’ education level as important control variables. Parents with higher family SES and education levels are more likely to provide more enriching learning activities and care, positively influencing their children’s emotions and behavioral problems (Conger and Donnellan, 2007). Additionally, family SES is considered a contributing factor to adolescent depression (Devenish, et al., 2017). For education level, no education or education up to high school was coded as 1, while college education or more was coded as 2. Family SES was measured on a five-point scale (“1 = Very poor,” “2 = Poor,” “3 = Neither poor nor rich,” “4 = Rich,” and “5 = Very rich”) in response to the question: “What is your family’s economic status?” This SES measure reflected respondents’ subjective perception of their family’s economic status. Furthermore, adolescents’ gender and grade level were included as control variables.

### 2.3 Analysis

The study model was tested using SPSS 26.0 software and the PROCESS Macro for SPSS developed by (Hayes, 2012). Descriptive statistics were computed for the study variables, and direct and indirect effects in the serial multiple mediation model were analyzed. Bootstrapping was employed to test the statistical significance of the mediating effects. The sample size was set at 5,000 with a 95% confidence interval to determine the significance of the mediating effect. Standardized regression coefficients were calculated by converting the variables to standardized scores for analysis, and statistical significance was set at 0.05.

## 3 Results

### 3.1 Basic statistics

Table 1 displays the correlation coefficients and descriptive statistics for the study variables. Notably, there were no strong correlations among the variables, which confirms a low risk of multicollinearity. Additionally, both kurtosis and skew-ness values were below ±2, and the Q–Q plot demonstrated a linear graph of the observed data, indicating a normal distribution.

**Table 1:** Descriptive statistics and correlation.

	1	2	3	4	5	6	7	8
1. Grade level	—							
2. SES	−0.05***	—						
3. education level (father)	−0.01	0.21***	—					
4. Education level (mother)	−0.01	0.21***	0.63***	—				
5. PSP	−0.11***	0.17***	0.17***	0.16***	—			
6. PR	−0.08***	0.06***	0.05***	0.05***	0.28***	—		
7. AD	0.17***	−0.14***	−0.18***	−0.18***	−0.21***	−0.17***	—	
8. Depression	0.10***	−0.12***	−0.02*	−0.03**	−0.18***	−0.26***	0.23***	—
M	0.49	3.00	4.22	3.84	3.39	2.64	2.55	2.08
SD	0.50	0.55	1.99	1.97	2.03	0.47	0.66	0.82
Skewness	0.05	−0.28	0.74	0.90	−0.01	−1.07	−0.07	0.98
Kurtosis	−2.00	3.03	−0.77	−0.36	−1.65	0.31	−0.24	1.31

SES, family socioeconomic status; PSP, parent-adolescent sports participation; PR, parent-adolescent relationship; AD, academic difficulty.

*p < 0.05, **p < 0.01, ***p < 0.001.

### 3.2 Estimation of the serial multiple mediator model paths

To analyze the serial multiple mediation effect of PR and AD on the relationship between PSP and depression, we employed Hayes (2012) PROCESS Macro, Model 6. Initially, we included the control variables SES, gender, and grade level and examined the effects of PSP on PR. The results revealed that the *R*^2^ was significant at 0.08 (*F*(6, 16,977) = 251.12, *p* < 0.001), and the standardized coefficient for the effect of PSP on PR was also significant at 0.27 (*t* = 36.00, *p* < 0.001, 95% CI [0.26, 0.29]). Furthermore, the effects of PSP and PR on AD were found to be significant, with an *R*^2^ of 0.13 (*F*(7, 16,976) = 346.41, *p* < 0.001). Specifically, the standardized coefficient for the effect of PSP on AD (*β* = −0.12, *t* = −15.90, *p* < 0.001, 95% CI [−0.14, −0.11]) and the effect of PR on AD (*β* = −0.11, *t* = −14.87, *p* < 0.001, 95% CI [−0.13, −0.10]) were both statistically significant. Finally, the effects of PSP, PR, and AD on depression were significant, with an *R*^2^ of 0.12 (*F*(8, 16,975) = 297.18, *p* < 0.001). The standardized coefficient for the effect of PSP on depression (*β* = −0.08, *t* = −10.35, *p* < 0.001, 95% CI [−0.10, −0.07]), the effect of PR on depression (*β* = −0.21, *t* = −27.38, 95% CI [−0.22, −0.19] *p* < 0.001), and the effect of AD on depression (*β* = 0.18, *t* = 22.83, *p* < 0.001, 95% CI [0.16, 0.19]) were all found to be statistically significant. Although the direct association between PSP and depression was statistically significant, the standardized coefficient was relatively small. This pattern suggests that the link between PSP and depression may be better understood by considering indirect pathways, particularly through PR and AD, rather than focusing only on the direct association (see Table 2).

**Table 2:** Dual mediation effect.

		PR(MV1)		AD(MV2)		Depression (DV)	
		Path	*β*	Path	*β*	Path	*β*
COV	Gender		0.03		−0.24***		0.09***
	Grade		−0.05***		0.14***		0.04***
	SES		0.02*		−0.09***		−0.08***
	Education level (father)		−0.01		−0.09***		0.05***
	Education level (mother)		0.01		−0.08***		0.02
IV	PSP	a1	0.27***	b1	−0.12***	b3	−0.08***
MV	PR			a2	−0.11***	b2	−0.21***
	AD					a3	0.18***
	R	0.29		0.35		0.36	
	R^2^	0.08		0.13		0.12	
		F (6, 16,977) = 251.12***		F (7, 16,976) = 346.41***		F (8, 16,975) = 297.18***	

SES, family socioeconomic status; PSP, parent-adolescent sports participation; PR, parent-adolescent relationship; AD, academic difficulty.

*p < 0.05, **p < 0.01, ***p < 0.001.

### 3.3 Evaluation of indirect effects

**Figure 2:**
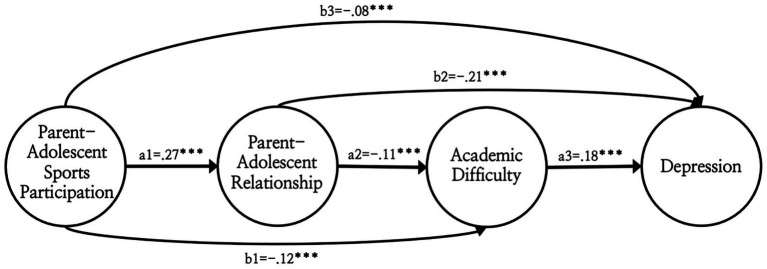
Serial multiple mediation model with standardized paths.

Figure 2 displays the analysis of the significance of the indirect effects in three paths using bootstrapping: (path 1: PSP → PR → depression, path 2: PSP → AD → de-pression, path 3: PSP → PR → AD → depression). The indirect effect in path 1 was estimated to be −0.06, and the 95% confidence interval did not include 0 (−0.06 to −0.05), indicating that the effect is significant. Similarly, the indirect effect in path 2 was estimated to be −0.02, and the 95% confidence interval did not include 0 (−0.02 to −0.01), con-firming its significance. Finally, the indirect effect in path 3 was estimated to be −0.01, and the 95% confidence interval did not include 0 (−0.006 to −0.004), confirming its significance. These results demonstrate that PSP has a significant effect on depression through the serial mediation of PR and AD.

To further examine the robustness of these serial mediation effects, additional analyses were conducted separately for boys, girls, lower-grade students, and higher-grade students. In all subgroups, the indirect effect in path 3 (PSP → PR → AD → depression) remained significant, with 95% confidence intervals not including 0 (boys: −0.005, CI [−0.006, −0.003]; girls: −0.01, CI [−0.008, −0.005]; lower-grade: −0.001, CI [−0.008, −0.005]; higher-grade: −0.004, CI [−0.006, −0.003]). These findings suggest that the serial mediation effect is generally consistent across gender and grade subgroups.

## 4 Discussion

First, the present study examined the path through which PSP is associated with depression in Chinese youth and found that PSP is linked to depression. This aligns with Kleppang et al.’s (2018) findings that sports activities involving social interactions are associated with diminished depression symptoms. Additionally, engaging in sports with parents fosters positive parent–child relationships and serves as a positive predictor of the parent–child relationship (Knoester and Randolph, 2019). A recent review further revealed that inadequate support from partners, friends, and family elevates the risk for depression and anxiety symptoms (Sufredini et al., 2022).

Second, the present study found that PSP is significantly associated with depression, with parent–child relationships (PR) being significantly mediated in this relationship. This aligns with earlier research suggesting that positive parent–child interactions can reduce depressive symptoms in adolescents (Mak et al., 2021; Chen and Harris, 2019). According to attachment theory (Fearon and Roisman, 2017), parent–child attachments play a crucial role in adolescent development. Engaging in sports with parents can strengthen secure attachments, leading to better psychological health. Conversely, adolescents with limited PSP may struggle to form secure attachments, facing difficulties in establishing stable and trusting relationships, which can lead to interpersonal problems and higher levels of depression (Ouyang and Liu, 2019). Therefore, fostering a close PR provides a warm familial environment, which may help mitigate adolescent depression.

Third, the present study found that perceived AD significantly mediated the association between PSP and depression. PSP may be associated with improved cognitive functioning, including memory and executive function, which could help adolescents cope more effectively with academic tasks (Kantomaa et al., 2016). At the same time, academic support derived from positive parent–child interactions may improve adolescents’ learning experiences and reduce their perceived AD (Zimmer-Gembeck et al., 2023). Lower levels of AD may further reduce feelings of helplessness, frustration, and negative cognitive patterns, which may in turn be associated with lower levels of depressive symptoms (Bu et al., 2025).

Finally, the association between PSP and depression is sequentially mediated by PR and AD. The positive relationships and family support formed through PSP may be associated with lower levels of perceived AD among adolescents (Spera, 2005). Conversely, if the family environment is characterized by discouragement and rejection, adolescents may perceive higher levels of AD, which could be associated with higher levels of depression. Therefore, these findings provide valuable insights into potential pathways for alleviating depression and the development of relevant PSP policies. However, further longitudinal research is needed to verify these associations.

In Figure 2, three direct effect paths (1. PSP → Depression, 2. PSP → AD, 3. PR → Depression) were significant, and the *R*^2^ values indicated that a portion of the variance in adolescents’ depression and related outcomes remained unexplained, suggesting the presence of potentially omitted variables. This is not surprising, as adolescent depression is a multifactorial outcome influenced by individual, family, academic, peer, and broader social factors. One possible additional mediator could be adolescents’ basic psychological needs. Sun et al. (2020) proposed that PSP could be a critical predictor of satisfaction of psychological needs. Parents play a pivotal role in adolescents’ cognitive, emotional, and behavioral development (Lin and Tsai, 2016). Positive PSP provides a favorable environment that meets adolescents’ psychological needs and promotes their well-being, while negative PSP may limit adolescents’ access to emotional warmth and social support (Brassell et al., 2016; Levin and Currie, 2010). Previous studies have shown that PSP has a positive impact on adolescents’ psychological needs (Niemiec et al., 2006; Tam et al., 2018). Hence, the satisfaction of adolescents’ basic psychological needs represents another potentially important factor in understanding the association between PSP and adolescent depression.

It is also important to consider potential bidirectional relationships between PSP and depression. While this study focused on the association from PSP to depression, it is possible that adolescents experiencing higher levels of depression may be less likely to participate in sports with their parents. Such a pattern could attenuate the observed associations, suggesting that the relationship between PSP and adolescent depression may be more complex than a simple unidirectional pathway. Future research using longitudinal or experimental designs is needed to examine these potential reciprocal effects, which would provide a more comprehensive understanding of how parental involvement in sports and adolescent mental health interact over time.

## 5 Conclusion

In conclusion, this study examined the association between PSP and depression in Chinese youth, focusing on the serial mediation of PR and AD. The findings suggest that PSP is associated with lower levels of depression, and this relationship may be indirectly linked through PR and AD. These results highlight the potential role of parent–adolescent relational and academic pathways in understanding how parent–adolescent sports participation relates to adolescent depression. However, due to the cross-sectional design, causal claims cannot be established, and the observed associations should be interpreted with caution.

### 5.1 Limitations

This study has several important limitations. First, the cross-sectional design precludes causal inferences between PSP, PR, AD, and depression, and future longitudinal or experimental studies are needed to establish causal relationships. Second, the measurement of key variables relied on brief or single-item indicators, which may limit the reliability and comprehensiveness of the assessment. Third, all data were collected via self-reports, which may introduce self-presentation and social desirability biases. Fourth, alternative models were not tested in this study, which limits the ability to compare different pathways or relationships among the variables. Future research should adopt longitudinal designs, multi-method assessments, and explore alternative models to strengthen the robustness and validity of the findings.

## Data Availability

Publicly available datasets were analyzed in this study. This data can be found at: http://ceps.ruc.edu.cn/.
